# Knowledge and awareness of dental implants among Syrian refugees: a cross sectional study in Zaatari camp

**DOI:** 10.1186/s12903-021-01806-7

**Published:** 2021-09-14

**Authors:** Nesreen A. Salim, Fatima Hafedh Meyad, Mariam Mohammad Al-Abdallah, Motasum Abu-Awwad, Julian D. Satterthwaite

**Affiliations:** 1 Prosthodontic Department, School of Dentistry, The University of Jordan Hospital, The University of Jordan, Amman, 11942 Jordan; 2 Bahcesehir University, Istanbul, Turkey; 3 Department of Paediatric Dentistry, Orthodontics, and Preventive Dentistry, School of Dentistry, The University of Jordan, Amman, Jordan; 4 Division of Dentistry, School of Medical Sciences, University of Manchester, Oxford Road, Manchester, M13 9PL UK

**Keywords:** Dental implants, Tooth replacement, Public dental health, Level of information, Syrian refugees

## Abstract

**Background:**

The popularity of implant dentistry is increasing dramatically, but the success of implant treatment depends on a patient’s knowledge and expectations.

**Methods:**

This study aimed to assess dental implant knowledge among refugees as a treatment option (n = 565), using face-to-face interviews. The frequency distribution of the responses in general and according to age, gender and education was calculated.

**Results:**

81.8% of the interviewees had missing teeth, however, only 26.2% replaced them. 16.6% of participants had never/hardly heard about implants. Females who never heard of implants were more than males (*P* < 0.001). 51.3% of participants described dental implants as a screw, and only 35.4% knew implants were placed in jawbones. 6.2% of respondents reported that implants required more care than a natural teeth, with 47.6% believing that diseases affect implant success. Friends were the main source of information (61.4%). Highly educated participants showed higher knowledge about implants.

**Conclusions:**

The surveyed sample revealed limited knowledge about dental implants with high cost being a major obstacle, warranting more strategies to increase awareness and to make implants more affordable for this population.

## Background

After the 2011 humanitarian crisis in Syria, over 5.6 million people have been internationally displaced into neighboring countries [1]. Jordan is one of the host countries for over three-quarters of a million Syrian refugees, of which 19% lives in camps. Zaatari camp is the largest refugee camp in Jordan, located close to Jordan’s northern border with Syria, since being established in 2012 it has become home for nearly 79,000 refugees [1]. Syrian refugees have suffered physical and psychosocial trauma, with repeated displacements, persecution, and limited access to healthcare services in poorly equipped temporary camps [2–4]. Sustained trauma and long-term deprivation has resulted in high health needs among refugees on arrival to their host countries [5].

Oral conditions remained a major public health challenge all over the world, posing a very serious public health challenge to policy makers [6]. The most frequent form of oral disease in refugees is dental caries, a public health problem that reduces the quality of life of individuals and communities worldwide [7]. Sadly, extraction has been reported to be the most common treatment for refugees in general and in Zaatari camp specifically [8, 9]. Tooth loss can lead to reduced function, drifting of adjacent teeth, supra-eruption of the opposing teeth, altered speech, loss of self-confidence, aesthetic problems, and feelings of bereavement [10–12]. Accordingly, restoring function and aesthetics is often a vital need for those patients [11].

Tooth loss is one of the main indicators of oral health in a population and between populations there are significant differences [12]. The prevalence of tooth loss was around 57% in a research conducted in India [13]. In the Eastern Province of Saudi Arabia, according to the Ministry of Health, 18,640 permanent teeth were extracted in 2019 [14]. It was also reported that the numbers of extracted permanent teeth increased from the year 2011 to 2015 in the country [14]. In another study, the prevalence, extent, and risk indicators for tooth loss were studied in a representative Brazilian population, showing that 94% of the subjects had experienced tooth loss [15]. In a recent review about oral health in European adults, Caries experience was extensive among adults (≥ 92%). In adults of 23 countries, the mean DMFT score ranged from 6.6 to 17.6 (median 12.1) [16]. In a study in Italy, the mean number of extracted teeth for each patient was 4.6 [12].

Many prosthetic options for replacing missing teeth are available, such as: removable partial dentures (RPD); fixed partial dentures (FPD); and dental implants [11, 17]. Multiple factors affect the choice of treatment modality replacement of missing teeth such as pain, dental phobia, unnecessary tissue damage to neighbouring teeth and cost [11]. Positive outcomes with dental implants have been confirmed [18, 19] and implant dentistry has become increasingly important in oral reconstruction [19–21]. However, due to misleading information patients might have increased or unrealistic expectations about their nature and performance [22–25].

In a previous study in Saudi Arabia, 6% of the patients were completely edentulous, 8% had single jaw edentulousness, and 74% were partially edentulous. Patients having class I and/or class II were treated most often with removable partial dentures (RPD), while patients having class III were treated with fixed partial dentures (FPD) [26]. A study from Mexico that included adults aged ≥ 18 years found that 6.3% were edentulous [27]. It was also found that 5% of UK adults between 55 and 64 years of age and 15% between 65 and 74 years are edentulous [28]. Moreover, the prevalence of edentulousness among US adults aged ≥ 15 years reached 4.9% in 2009–2012 [29]. The prevalence of edentulousness in Ghana was reported to be 2.8% among people aged ≥ 50 years and 1.3% among adults aged 65 years or more in Ibadan Nigeria [30, 31].

Implants may sometimes be the only option for successful rehabilitation to restore quality of life of patients with significant maxillo-facial defects such as those sustaining trauma in war [32, 33]: this is particularly relevant for refugee populations who have a high risk category of becoming war-injured [22]. The functional results of osseointegrated implants used for rehabilitation of war-injured patients are favourable and comparable to their application in otherwise normal edentulous patients [32].

Lack of knowledge about treatment options presents a challenge, especially in developing countries [34]. In a camp setting with limited services and resources, there is dearth of information regarding patients’ knowledge of tooth replacement, including the use of implants as an option to replace missing teeth [34], with unrealistic expectations, and negative views towards implants being common [23, 32]. It has been found that there is a significant relationship between comfort rating and “how well informed” patients are [25]. In general terms, a greater understanding of patients’ knowledge and expectations allows for more appropriately directed treatment plans to be formulated, and the context of this population group would allow for better planning of services, both practically and fiscally [25].

The aim of this study was to assess the awareness of refugees and war victims regarding dental implants as a treatment option among refugees (data not hitherto available), their source of information, and attitude regarding the use of dental implants as a treatment option compared with other conventional treatment modalities; to allow healthcare providers to design suitable communication strategies for this population group.

## Methods

### Ethical approval

The research protocol was approved by the Ethical Committee of the Faculty of Dentistry of the University of Jordan. Written informed consent was obtained from participants. All the participants were informed regarding the objectives and aims of the study.

### Study group and design

This survey was conducted between June and November 2019, during which period 565 adult (over 18) Syrian refugees attending Zaatari dental clinic were interviewed. All participants were registered as refugees in Jordan and residing in Zaatari camp. The age, gender, education level and oral hygiene practices for the interviewees were recorded: participants were stratified into 4 age categories: 18–29 years, 30–39 years, 40–49 years and 50 years of age and above. Education levels were: low (none, primary school), moderate (secondary school), and high level (college and university).

A face-to-face based questionnaire was designed, based on previous studies [23, 24]. The questions were revalidated by an experienced prosthodontist (N A.S.), and a pilot study was performed on 25 subjects to evaluate the questionnaire. The questionnaire was modified accordingly. The final questionnaire comprised demographic details and 20 close-ended multiple-choice questions to assess patient awareness and perceived cost of dental implants. The following aspects were recorded:

1. Dental status and awareness about tooth replacement: including self-reported dental status, importance of replacing missing teeth if any, general attitude towards the need for prosthodontic treatment and the different alternatives for replacing the missing teeth.
2. Level of information about dental implants: assessing awareness (subjective) and level of information about dental implants as a prosthetic option (objective).
3. Dental implant information sources and subjectively perceived need for information.

All participants were interviewed in a private setting in Zaatari dental clinic by one interviewer (a prosthodontist: N A.S.). In order to reduce potential bias, the interviewer was blinded to demographic data of the interviewee, with this data being recorded separately by two intern dentists using paper based questionnaires.

### Sample size calculation

The sample size was calculated using the following formula for cross-sectional studies [35]:

$${\text{n}} = {\text{Z}}^{{2}} {\text{P}}\left( {{1} - {\text{P}}} \right)/{\text{d}}^{{2}}$$

where n = sample size, Z = 1.96 (level of confidence 95%), *P* = 0.5 (expected proportion in population) and d = 0.05 (precision).

$${\text{n}} = \left( {{1}.{96}} \right)^{{2}} \times 0.{5}\left( {{1} - 0.{5}} \right)/\left( {0.0{5}} \right)^{{2}} \quad \left( {{\text{n}} = {384}.{16}} \right)$$

This is the sample size if we assume the population number is infinite, given Zaatari camp has population of 76.989, then the following formula was used to determine the required sample size:

$$\begin{aligned} & {\text{Sample}}\;{\text{size}} = {\text{n}}/{1} + \left[ {\left( {{\text{n}} - {1}} \right)/{\text{population}}} \right] \\ & \quad = {382}\left( {{\text{this}}\;{\text{is}}\;{\text{the}}\;{\text{required}}\;{\text{sample}}\;{\text{size}}\;{\text{at}}\;{95}\% \;{\text{of}}\;{\text{confidence}}\;{\text{level}}} \right). \\ \end{aligned}$$

Data collection was planned with a sample size of 565 refugees to allow for drop-outs and non-participation and to provide sufficient power for the detection of statistically significant differences between sub-groups based on gender, age and level of education.

### Statistical analysis

Data were analyzed using SPSS version 23.0 (Armonk, NY: IBM Corp., 2015). Descriptive statistics and frequency tables were created and results presented as percentages and proportions. Tests of association between the independent variables of gender, age group, and level of education, and the variables measuring the awareness and knowledge of dental implants were conducted using chi square statistics. For comparisons involving more than two groups, significant differences between tested groups were explored using Post Hoc testing. For comparison of the education on the objective knowledge on implants, Kruskal–Wallis H test was used. The level of significance was set at *P* < 0.05.

## Results

A total of 565 participants were interviewed. The demographic details, education level and oral health practices are presented in Table 1.

**Table 1:** Demographic characteristics and oral health profile of the population

Demographic variables	Frequency (N=565)	Percent (100%)
*Gender*		
Male	231	40.9
Female	334	59.1
*Age*		
A1 (18–29 years)	178	31.5
A2 (30–39 years)	195	34.5
A3 (40–49 years)	119	21.1
A4 (50 years or more)	73.0	12.9
*Education*		
No education	49.0	8.6
Primary school	300	53.1
High school	141	25.0
College	32.0	5.7
University	43.0	7.6
*Oral health care*		
None	76	13.5
Occasionally	172	30.4
Once a day	221	39.1
Twice a day	96.0	17.0

### Dental status and awareness about tooth replacement

The dental status and awareness of tooth-replacement options are given in Table 2. At least one tooth was lost by extraction in 81.8% of our sample (58.2% were females, 41.8 were males), with caries being the most common reason for extraction (87.7%). 26.9% lost their teeth recently (2–3 years ago) and 16.6% lost their teeth 4–7 years ago. Participants with low level of education had significantly more missing teeth (*P* < 0.001)*.*

**Table 2:** Dental status and knowledge of prosthetic options

Questions: Awareness of tooth replacement	Total	Gender %		Age group %				Education level %		
	%	M	F	A1	A2	A3	A4	ED1	ED2	ED3
Do you have missing teeth?										
Yes	81.8	83.5	80.5	66.9*	83.6	91.6*	97.3*	87.7*	75.2	66.7*
Did you replace your missing teeth?										
Yes	26.2	31.6	23.0	10.4*	25.0	41.2*	26.2	24.5	27.3	37.9
Do you think the replacement of missing teeth is important?										
Very important	86.9	85.3	88	83.1	87.7	89.9	89.0	86.2	86.5	90.7
Somewhat important	9.7	10.8	9.0	11.8	10.8	5.9	8.3	10.0	9.9	8.0
Not important at all	3.4	3.9	3.0	5.1	1.5	4.2	2.7	3.7	3.5	1.3
What is the reason for replacing missing teeth?										
Esthetic	8.8	8.7	9.0	10.7	7.7	10.1	5.5	7.4	14.2	5.3
Functional	51.5	52.4	50.9	46.1	48.2	52.9	71.2*	58.2	44.0	34.7
Both esthetic and functional	39.6	39.0	40.1	43.3	44.1	37.0	23.3*	34.4	41.8	60.0
Do you know the different ways of replacing missing teeth										
Yes	80.7	80.1	81.1	75.3	83.6	77.3	91.8	82.5	78.7	76.0
Are you aware of fixed partial dentures?										
Yes	81.2	82.3	80.5	74.7	84.6	79.8	90.4	81.7	82.3	77.3
Are you aware of removable dentures?										
Yes	79.8	81.4	78.7	71.3*	82.1	82.4	90.4	81.7	76.6	77.3
Have you ever heard about dental implants?										
Yes, very well	83.4	86.1	81.4	82.6	84.6	85.7	78.1	80.8	87.9	86.7
Yes, poorly	8.1	9.1	7.5	9.6	7.7	9.2	4.1	9.2	5.0	9.3
Not at all	8.5	4.8*	11.1*	7.9	7.7	5.0	17.8	10.0	7.1	4.0

*Significance level at *P* < 0.01 within each group in the same row

M: males, F: females. ED1: none and primary education, ED2: secondary, ED3: college and university. A1: age 18–29 years, A2: 30–39 years, A3: 40–49 years, A4: ˃50 years

As reported frequency of brushing increased, the percentage of participants with missing teeth decreased: 93.4%, 83.1%, 80.5% and 72.9% for none, occasional, 1/day and 2/day brushing respectively. Brushing frequency increased with education level: those not brushing was 16.3% of participants with low education level, 9.9% of moderately educated level and 6.7% of highly educated level, and those brushing twice a day in each level of education were: 13.5% with low education level, 18.4% in moderate level and 30.7% of the group with high level of education.

Participants who were 50 years of age or older cited functional reasons for tooth replacement significantly more than other age groups (*P* < 0.001) and aesthetic reasons less than the other age groups (*P* < 0.001, Table 2). No significant difference was detected between levels of education regarding how important it was to replace missing teeth.

Knowledge regarding alternatives for replacing missing teeth varied (Table 2), with awareness of removable dentures significantly lower in the younger age group (*P* < 0.001) and significantly more females had never heard of implants compared to males (*P* < 0.001).

### Knowledge of dental implants

Many participants could not describe an implant despite being aware of them as an option (Table 3). The number of females correctly described implants was significantly lower than males (*P* < 0.001) and females who could not describe the implant at all were significantly more compared to males (*P* < 0.001, Table 3). The majority who never heard about implants were of low level of education: 71.9% compared to 5.3% who were highly educated.

**Table 3:** Level of information about dental implants

Questions: Awareness of dental implant therapy	Total%	M%	F%
	(n = 565)	(n = 231)	(n = 334)
How would you describe a dental implant?			
Screw	51.3	64.9*	41.9*
Piece of metal	2.8	3.0	2.7
Heard about it, but cannot explain	35.8	27.3*	41.6*
Never heard about it	10.1	4.8*	13.8*
How long do you think an implant lasts?			
For a life time	31.2	36.8	27.2
(5–10) years	10.6	13.0	9.0
˃ 10 years	6.0	5.2	6.6
Not sure	52.2	45.0*	57.2*
Where in the jaw do you think implants are anchored?			
In the jawbone	35.4	50.2*	25.1*
In the gum	30.8	30.3	31.1
In neighboring teeth	3.5	1.7	4.8
Do not know	30.3	17.7*	38.9*
In your view, up to which amount you need to pay for an implant?			
145USD	5.7	5.2	6.0
283-425USD	10.8	13.4	9.0
565-710USD	14.5	17.7	12.3
850-990USD	9.4	8.7	9.9
≥ 1400USD	17.7	19.4	16.5
Do not know	41.9	35.5	46.4
Are you aware of medical problems that may interfere/lower the success rate of dental implant?			
Yes	47.6	51.5	44.9
No	52.4	48.5	55.1
If yes n = 269, what are the medical conditions contributing to the failure of an implant?			
Bone disease	30.9	35.3	27.3
Cardiac disease	32.7	28.6	36.0
Diabetes	71.7	78.2*	66.7*
Cancer	13.8	16.8	11.3
Gingival inflammation	9.7	8.4	10.7
Do you think implants need special care and oral hygiene compared to natural teeth?			
No, are cleaned like natural teeth	44.2	42.9	45.2
No, need less care than natural teeth	31.2	32.5	30.2
Yes, need more care than natural teeth	6.2	7.8	5.1
Does not need any special care	3.2	3.0	3.3
Do not know	15.2	13.9	16.2
What is the reason for not considering dental implant therapy?			
Perceived no need to replace teeth	15.6	14.3	16.5
Fear from surgery	2.5	1.3	3.3
High cost	73.6	78.4*	70.4*
Lack of understanding of the nature of the procedure	3.4	2.6	3.9
Lack of knowledge as not given information from the dentist	7.1	4.3*	9.0*

*Significance difference at *P* ˂ 0.01 between variables in the same group, in the same row

M: males, F: females

Knowledge of implant lifespan is given in Table 3. More females were not sure about how long implants could last than males (*P* < 0.001). Level of education or age had no significant effect on correct knowledge of lifespan of an implant (*P* ˃ 0.05), although interestingly more ‘highly educated’ (49.3%) than ‘low educated’ (28.9%) thought implants lasted for a lifetime.

Knowledge regarding placement of implants in the jawbone is also given in Table 3. Overall 35.4% had correct knowledge, with correct knowledge being significantly higher in Males (*P* < 0.001). This was also significantly higher in the A3 (40–49 years) age group (*P* < 0.001, Table 4), but lower for ‘low educated’ participants (*P* < 0.001, Table 4).

**Table 4:** Selected implant knowledge according to education and age

Questions	Education %			Age %			
	ED1	ED2	ED3	A1	A2	A3	A4
How would you describe a dental implant							
A: Screw	49.3	48.9	65.3	47.8	56.9	53.8	41.0
How long do you think an implant lasts?							
A: More than 10y	9.5	14.2	9.3	11.8	7.7	13.4	11.0
Where in the jaw do you think implants are anchored?							
A: In the jaw bone	30.1*	41.8	48.0	38.2	28.7	47.9*	26.0
Are you aware of medical problems that may interfere with the success rate of dental implant?							
A: Yes	41.5*	51.8	68.0*	42.7	48.2	57.1	42.5
Do you think implants need special care compared to natural teeth?							
A: Yes, need more care than natural teeth	43.0	41.8	54.7	40.4	51.8	41.2	38.4
In your view, up to which amount you need to pay for an implant?							
A: 850-990USD	10.3	7.8	8.0	7.9	11.8	9.2	6.8

*Significance difference at P˂0.01 between variables in the same group, in the same row

ED1: none and primary education, ED2: secondary, ED3: college and university. A1: age 18–29 years, A2: 30–39 years, A3: 40–49 years, A4: ˃ 50 years

Few participants were aware of costs of implant treatment although there were no significant differences were reported according to education level, age or gender (Tables 3 and 4, *P*˃0.05).

Knowledge of the interplay of disease with success of implants (Table 3) was significantly greater for ‘highly educated’ (*P* < 0.001) with those with a low level of education being significantly less aware of this *(P* < 0.001, Table 4). More males stated that diabetes might contribute to failure of implants than females (*P* = 0.03), and unsurprisingly most diabetic patients in this sample reported that diabetes affects implant success rate (86.2%).

By far, the main reason given for not considering implant therapy was high cost (72.2%), with this being significantly higher in males than females (*P* = 0.03), and lack of knowledge as a reason was significantly higher in females than males (*P* = 0.03). In both the low and highly educated groups, implant treatment was reported to be too expensive (74.7% for both).

When the participants were ranked according to the total score for correct answers for the five questions reflecting their knowledge objectively (what is an implant, where is it placed, survival rate, hygiene care and effect of disease on success rate), only 1.9% were extremely knowledgeable (5/5), 10.4% moderately knowledgeable (4/5), 22.3% and 24.1% somewhat (3/5), 22.7% slightly knowledgeable and 18.6% not at all knowledgeable.

### Sources of information and subjectively perceived need for information

The main source of information and want for further information is given in Table 5. Significantly more females (68.3%) relied on friends for knowledge compared to 51.5% males (*P* < 0.001), although this was significantly less for those with a high level of education (*P* < 0.001). Those with a low level of education used the internet to a lesser extend (13.5%) than those with a higher level of education (34.7%) with the difference being significant (*P* < 0.001): this was also significantly greater for males (28.1%) compared to females (9.9%) (*P* < 0.001, Table 5), but not significantly different based on age groups, using the dentist, friends, magazine, radio (*P* = 0.52, 0.35, 0.73, 0.402, 0.36) respectively.

**Table 5:** Sources of information about dental implants

Questions: Sources of information and subjectively perceived need for information	Total%	Gender%		Education%		
	(n = 565)	M (n = 231)	F (n = 334)	ED1	ED2	ED3
What are your sources of information about dental implants?						
Dentists	21.8	24.2	20.1	22.1	21.3	21.3
Friends and acquaintance	61.4	51.5*	68.3*	65.0	63.8	40.0*
Internet sources	17.3	28.1*	9.9*	13.5*	17.7	34.7*
Radio and TV	7.1	6.9	7.2	6.6	6.4	10.7
Newspapers and magazines	1.2	0.9	1.5	1.4	0.0	2.7
Would you like to know more about dental implants?						
Absolutely	60.4	58.4	61.7	57.3	63.8	68.0
Yes, just a little	20.5	21.2	20.1	21.2	19.1	20.0
Never	19.1	20.3	18.3	21.5	17	12.0

*Significance difference at *P* ˂ 0.01 between variables in the same group, in the same row

M: males, F: females. ED1: none and primary education, ED2: secondary, ED3: college and university

## Discussion

Oral health status of refugee population compares poorly with that of other populations [2, 36], with a very high level of unmet oral health needs.

Refugee populations, such as at Zaatari camp, are at high risk of having very challenging clinical situations, with war-injured victims left with maxillofacial defects: in such cases implant therapy may be the only reconstructive option to rehabilitate and to improve their quality of life [32], yet there is a lack of data concerning attitudes of this group towards tooth replacement and different treatment options including dental implants. Moreover, the high number of people in the current study with missing teeth (81.8%) highlights the high demand for replacement of missing teeth and the importance of knowledge about different prosthetic options.

Although tooth loss can be prevented, its incidence has not declined in recent decades and it is still considered a public health care issue [6]. This study reported a very high prevalence of tooth loss in refugee population with caries being the most common reason for extraction. High dental disease burden is extremely correlated to accessibility, income, availability of dental services and the perception of necessity of regular dental care; all of these factors are major issues in refugee populations and may explain the high prevalence of tooth loss [2, 3, 8, 9].

Although the majority of the respondents reported that replacing missing teeth is very important, only 20% had replaced their missing teeth. This could be related again to the challenging situations and the extreme limitations in service availability in camps [2, 8]. Other studies have concluded that the high cost, low felt need for treatment and fear of painful dental procedures may delay dental treatment being sought [4, 9, 37].

In this study, the majority of patients wanted their lost teeth restored primarily for better mastication. While most patients are familiar with both fixed and removable prosthodontic alternatives, they are less knowledgeable with implant supported prostheses. In line with our findings, in a previous research, 76.2% of the study group was opined that the missing teeth should be replaced by prosthetic means and the majority were keen in getting them replaced mainly for the comfort in mastication [17]. However in that study, although 77.9 and 32.9% were aware of the removable prostheses and implants respectively, only 25.2% knew about tooth supported bridges as an option of replacement of missing teeth [17]. Additionally, patients with Kennedy class I and II showed the highest overall demand for prosthetic replacement of missing teeth confirms that patients’ concern for the improvement of mastication plays an important role in this high demand [17].

Importantly in the current study, more missing teeth were related to low level of education and weak oral hygiene practices. These results were in line with previous findings, as there was a social gradient in tooth loss by education and their results showed that living in disadvantaged municipalities cannot overcome the risk associated with low schooling [38]. Moreover, it has been reported that experience of dental caries was associated with refugee behaviors, including poor dental hygiene methods and level of education, where higher education levels provided a better understanding of oral and general health [2]

Previous studies assessing awareness of dental implants have shown high levels (77.0% and 70.1% in American and a Norwegian samples, respectively [39, 40]), similar to the present study (83.4%); however, this is subjective awareness, and when the actual knowledge was tested (objective awareness), only 1.9% were extremely knowledgeable and 10.4% had moderate knowledge about dental implants. This is in comparison to a Saudian sample where 28.1% of participants were well informed about dental implant as a treatment modality for teeth replacement [41]. Such variation of awareness levels among different studies reflects differences between the era when the study was conducted, the population group studied, education level, and the age range of the study sample [12].

Male gender, high professional qualifications, and age (40–49 years) were the main factors associated with higher level of information about implants. This is in line with previous similar studies [23, 40]. This could be attributed to increased interest and awareness of advancement in dental technology among the middle aged and educated generation. The important role that the dentists should take to explain procedures carefully and comprehensibly to their potential patients is highlighted by the 7.1% of participants who did not consider implant as an option because of lack of knowledge.

The high cost of prosthetic treatment and the perceived no need to treatment, limit the accessibility of refugees to proper dental services in their home countries and hosting countries [5, 42]. As seen in previous studies [17, 43] and the current study, cost factor was the major disincentive to implant dentistry. Thus, considering that the costs of the treatment to be covered by NGOS (such as Doctors without borders) would definitely raise the willingness of patients, who are in high need, for receiving the dental implant treatment.

Dentists have been reported as the main source of information about dental implants [41, 43, 44], along with media sources [40]. However, in the present study friends were the main source of information followed by dentists—this has also been reported in a previous survey conducted in Saudi Arabia [45]. In the present study, the reduced number of dentists and the limited access to dental care in camps explains why they played a secondary role.

This percentage of those interviewed who cited the jawbone as the host site for implants compares poorly with previously reported results (61%) [23] as do results on aftercare for dental implants: those thinking implants required less care being 31.2% compared to 4.0–13.5% [24, 45]. However, the percentage of participants who could not describe dental implants correctly is comparable to those reported previously [46].

Adults with uncontrolled or poorly controlled diabetes are at threefold periodontal risk compared to non-diabetics and diabetes has been frequently reported to be associated with bone metabolic and osteopathic changes [47]. Accordingly, it is crucial that potential patients be aware of the implications of diabetes on the outcomes of implant therapy. A high number were aware of medical problems affecting the success rate of dental implants (higher in ‘highly educated’ participants), similar to that reported (85.0%) in a study conducted in Saudi Arabia [48]. However, these are higher than those reported in a study conducted on a Jordanian population, where that only 48% of the sample was aware that diabetic patients were more prone to oral diseases [47].

Although face-to-face interviews were time-consuming and cost-intensive, it offers many advantages over mail and telephone surveys in terms of the complexity and quality of the data collected and it produces response rate of 100% [23]. Published studies based on mailed or hand-out questionnaires tend to produce unsatisfactory low response rates, which were reported to be major drawbacks of these surveys [40, 49]. For example, a British group recorded a response rate of 66.9% compared to 100% response rates in face to face survey-based studies [43, 44, 50].

The increased cost as well as the additional potential sources of response bias are main drawbacks of face-to-face surveys. Nevertheless, this problem can be addressed by providing interviewers the necessary training and practice, to ensure that they are collecting the data without introducing bias, and do not, through their words or actions, unintentionally influence respondents to answer in a specific way [51]. In this study, information was collected on 20 items, which exceeded the number of items covered in most other surveys, considering age, gender and education as distinguishing factor [23, 40, 41, 46, 48].

## Conclusions

Increased awareness among patients regarding dental implant procedures can help in eliminating any negative preconceptions that may have been caused due to the lack of adequate communication and knowledge. Evidence-based information about the potential for, and limitations of, oral implants depends on coordinated communication: future strategies should be targeted to more professional public relations and patient information. The development of further dental services, including implant provision, would be of benefit to disadvantaged populations with a high need for such implant treatment for rehabilitation: the needs of refugee populations are many (and often basic), nonetheless, the role of oral rehabilitation and its impact on quality of life and general well-being should not be overlooked in considering strategic and financial planning of care and support.

## Data Availability

All collected data from patients analyzed during this study are included in this published article. The datasets used and analyzed during the current study are available from the corresponding author on reasonable request.

## Abbreviations

H: Kruskal–Wallis test
FPD: Fixed partial dentures
RPD: Removable partial dentures
